# RNA based individualized drug selection in breast cancer patients without patient-matched normal tissue

**DOI:** 10.18632/oncotarget.25981

**Published:** 2018-08-17

**Authors:** Michael Forster, Adam Mark, Friederike Egberts, Elisa Rosati, Elke Rodriguez, Martin Stanulla, Dirk Bauerschlag, Christian Schem, Nicolai Maass, Anu Amallraja, Karla K. Murphy, Bruce R. Prouse, Raed A. Sulaiman, Brandon M. Young, Micaela Mathiak, Georg Hemmrich-Stanisak, David Ellinghaus, Stephan Weidinger, Philip Rosenstiel, Norbert Arnold, Brian Leyland-Jones, Casey B. Williams, Andre Franke, Tobias Meißner

**Affiliations:** ^1^ Institute of Clinical Molecular Biology, Kiel University, Kiel, Germany; ^2^ Current address: Center for Computational Biology and Bioinformatics, Department of Medicine, University of California, San Diego, CA, USA; ^3^ Department of Dermatology, Schleswig-Holstein University Hospital, Kiel, Germany; ^4^ Department of Pediatric Haematology and Oncology, Hannover Medical School, Hannover, Germany; ^5^ Department of Gynaecology and Obstetrics, Schleswig-Holstein University Hospital, Kiel, Germany; ^6^ Mammazentrum Hamburg, Hamburg, Germany; ^7^ Department of Molecular and Experimental Medicine, Avera Cancer Institute, Sioux Falls, SD, USA; ^8^ Pathology, Avera McKennan, Sioux Falls, SD, USA; ^9^ Department of Pathology, Schleswig-Holstein University Hospital, Kiel, Germany

**Keywords:** breast cancer sequencing, normal RNA expression, drug selection, formalin fixed paraffin embedded, fresh frozen

## Abstract

**Background:**

While standard RNA expression tests stratify patients into risk groups, RNA-Seq can guide personalized drug selection based on expressed mutations, fusion genes, and differential expression (DE) between tumor and normal tissue. However, patient-matched normal tissue may be unavailable. Additionally, biological variability in normal tissue and technological biases may confound results. Therefore, we present normal expression reference data for two sequencing methods that are suitable for breast biopsies.

**Results:**

We identified breast cancer related and drug related genes that are expressed uniformly across our normal samples. Large subsets of these genes are identical for formalin fixed paraffin embedded samples and fresh frozen samples. Adipocyte signatures were detected in frozen compared to formalin samples, prepared by surgeons and pathologists, respectively. Gene expression confounded by adipocytes was identified using fat tissue samples. Finally, immune repertoire statistics were obtained for healthy breast, tumor and fat tissues.

**Conclusions:**

Our reference data can be used with patient tumor samples that are asservated and sequenced with a matching aforementioned method. Coefficients of variation are given for normal gene expression. Thus, potential drug selection can be based on confidently overexpressed genes and immune repertoire statistics.

**Materials and Methods:**

Normal expression from formalin and frozen healthy breast tissue samples using Roche Kapa RiboErase (total RNA) (19 formalin, 9 frozen) and Illumina TruSeq RNA Access (targeted RNA-Seq, aka TruSeq RNA Exome) (11 formalin, 1 frozen), and fat tissue (6 frozen Access). Tumor DE using 10 formalin total RNA tumor samples and 1 frozen targeted RNA tumor sample.

## INTRODUCTION

Breast cancer is the most common cancer affecting women, with over 265,000 newly diagnosed cases in the USA [1] and over 70,000 in Germany [2], respectively. On initial diagnosis, patients are treated according to the histological, molecular and in some cases even genetic classification of their cancer. Routine classification in the US and Germany comprises tumor size and lymph node involvement, as well as immunohistological staining for the estrogen, progesterone and Her2 receptors. Tumor tissue based DNA testing with targeted next-generation sequencing panels is additionally performed in a growing number of centers. Genetic counselling and blood tests for hereditary breast cancer risk variants in *BRCA1, BRCA2, TP53* and other genes are also routinely offered. Standard treatment with curative intent contains surgical resection of the tumor (segmentectomy or mastectomy) and lymph node staging. Patients with hereditary risk variants in *BRCA1, BRCA2* or other core breast cancer risk genes may be offered bilateral subcutaneous mastectomy and ovarectomy. Adjuvant treatment with drugs and/or radiotherapy follows, depending on many factors including staging, menopausal status and molecular findings.

RNA expression profiling of breast cancer FFPE samples is well established to classify patients into low risk, intermediate risk and high risk groups [3] and has been introduced into the standard of care guidelines in some countries as e.g. a 21-gene real-time PCR assay (Oncotype DX, Genomic Health). More recently, whole exome and whole transcriptome sequencing of tumor versus normal tissue is increasingly being considered to help guide drug selection, especially for aggressive forms of breast cancer, metastatic breast cancer, and recurrences [4, 5]. Whole exome sequencing can inform of actionable mutations, tumor mutation burden and pharmacogenomic variants. Whole transcriptome sequencing with paired-end reads can additionally inform of actionable fusion genes, expressed mutations (especially neoantigenic mutations), and loss or gain of gene expression compared to healthy normal tissue. However, currently, patient-matched normal breast tissue is not routinely asservated and therefore not routinely available when needed, especially when the patient experiences a relapse after bilateral mastectomy [5]. Even when a patient-matched sample of normal breast tissue is available, additional healthy samples are required to distinguish normal gene expression variability in healthy breast tissue from pathological gene expression in tumor tissue. To address these problems and allow RNA based differential expression analysis to be carried out for breast cancer patients with or without patient-matched healthy breast tissue, we collected healthy normal ductal tissue from breast reduction operations and from resected healthy tissue that was adjacent to tumor tissue. We focused on two specific RNA sequencing library preparation kits because each is suitable for the small amounts of RNA that can be recovered from fine needle aspirates. The Roche KAPA stranded RNA-Seq kit with RiboErase worked well for most samples that we tried with 50 to 100 ng of RNA. The Illumina TruSeq RNA Access kit worked well for all samples that we tried, with only 10 to 100 ng of RNA.

**Figure 1:**
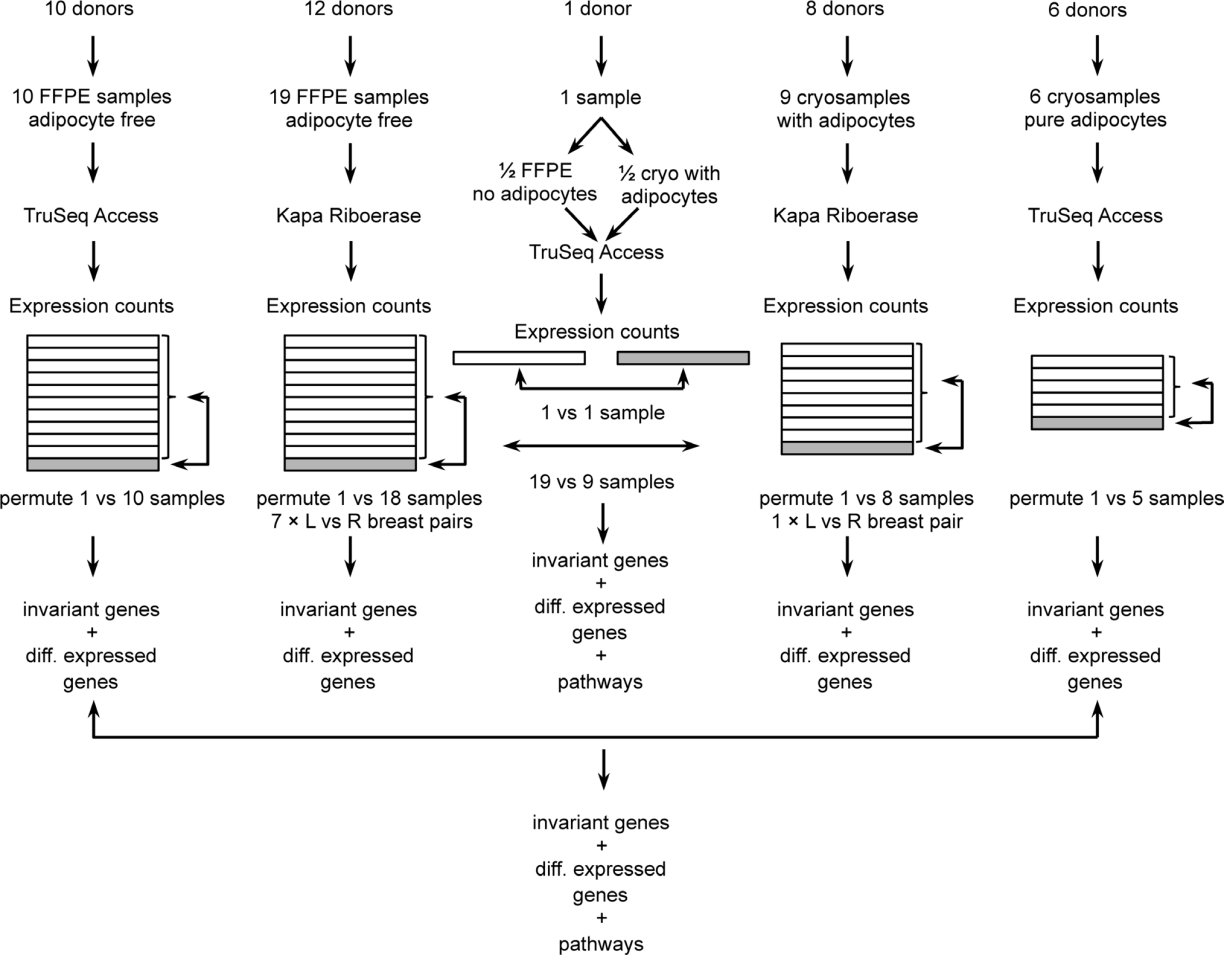
Overview of samples, RNA-Seq and data quality control

It is important to note that RNA expression values obtained with two different kits should not be mixed into the same differential expression analysis, because each kit may introduce its own biases. Specifically, the Roche KAPA RNA RiboErase kit is used to generate sequencing libraries that, in sum, cover the entire transcript and also include non-coding genes. In contrast, the Illumina TruSeq RNA Access kit protocol is used to generate targeted RNA sequencing libraries. The TruSeq RNA Access protocol includes random primer amplification and exome baits to capture just the protein-coding regions of protein coding genes. It should further be noted that the asservation method may introduce some differential expression bias. We therefore generated healthy normal breast ductal tissue reference expression data from formalin fixed paraffin embedded samples as well as from fresh frozen samples (see Figure 1). For quality control, we performed extensive differential expression analyses as summarized in Figure 1, (a) within each group of healthy samples, (b) between groups of healthy samples, (c) between patient-matched pairs of left and right healthy breast tissue, and (d) between tumor and healthy samples. After we identified adipocyte signatures in the fresh frozen breast ductal tissue samples, we investigated the extent by which fat cells in the healthy tissue may confound known drug-gene associations. Therefore, we also sequenced a batch of fresh frozen pure fat tissue samples, and a single breast ductal tissue sample that we split into a fresh frozen piece (macro-dissected from fat tissue, but not entirely without fat tissue) and a formalin-fixed piece (laser-micro-dissected).

We annotated the quality-controlled normal healthy breast ductal tissue data with coefficients of variation. Additionally, we annotated genes that were highly differentially expressed between patient-matched left and right breast tissue pairs, or between tissues with and without adipocytes. These annotations allow the highly variable genes to be identified or excluded from differential expression analysis between patient tumor and our normal tissue data. Table 1 summarizes the numbers of genes with low gene expression variability in healthy tissue (log2 of fold change less than or equal to 2), depending on kits and asservation methods. Supplementary Tables 1–5 detail the quality-controlled genes and their annotation. Supplementary Tables 6–9 summarize the immune repertoire statistics of T-cell receptors (TCR) and B-cell receptor immunoglobulins (IG). Our reference data will allow personalized differential expression and immune repertoire analyses to be performed for breast cancer patients who have no remaining healthy breast tissue, with the aim of matching the differential expression data to suitable drugs.

**Table 1:** Number of genes with low expression variability in healthy breast ductal tissue

Asservation	RNA-Seq	Samples	All genes	BrCa genes	Cancer biomarker	BrCa biomarker	Supp table
FFPE	KAPA	19	10259 (43%)	250 (48%)	118 (64%)	50 (65%)	1
FF	KAPA	9	6720 (28%)	147 (28%)	86 (47%)	38 (49%)	2
FFPE vs FF	KAPA	19 vs 9	6465 (27%)	143 (28%)	83 (45%)	36 (47%)	3
FFPE	Access	10	6981 (29%)	204 (39%)	110 (60%)	49 (64%)	4
FFPE (epi) vs FF (fat)	Access	10 vs 6	3038 (13%)	98 (19%)	54 (30%)	21 (27%)	5

Percentages are given with respect to 23686 RefSeq genes, 518 BrCa associated genes, 183 Cancer biomarker genes, or 77 BrCa biomarker genes, respectively. FFPE (epi) formalin fixed paraffin embedded epithelial ductal breast tissue, FF (fat) fresh frozen pure fat tissue.

## RESULTS

### Overview

We generated the healthy normal ductal breast tissue expression data from 20 formalin fixed paraffin embedded tissue biopsies (FFPE) and 10 fresh frozen (FF) tissue biopsies. These included 7 pairs of left and right breast tissue samples from the same patients. For comparison, we added 10 breast cancer tissue biopsies and 6 pure fat tissue biopsies. Sequenced read counts per million (CPM) are available in Supplementary Table 10. To compare our fat sample findings from our targeted RNA-Seq sequences with previously available data, we also analysed GTEx mRNA-Seq expression data from subcutaneous fat (*n* = 442) and breast tissue (*n* = 290) (https://www.gtexportal.org).

### Sequencing statistics

Healthy tissue samples were sequenced with 40 million and 60 million paired-end 75 base-pair reads per frozen and formalin sample, respectively, using the Roche Kapa RiboErase RNA-Seq kit, and 50 million reads per formalin sample using the Illumina TruSeq RNA Access kit (detailed numbers in Supplementary Table 11). Tumor samples were sequenced with 60 to 80 million reads, and the pure fat samples were sequenced with 20 million reads. The alignments to hg19 with RefSeq annotation for 23686 genes and subsequent filtering of low count genes resulted in 16808 genes that were useable for differential expression analysis between normal and tumor tissue.

### Principle component analysis

**Figure 2:**
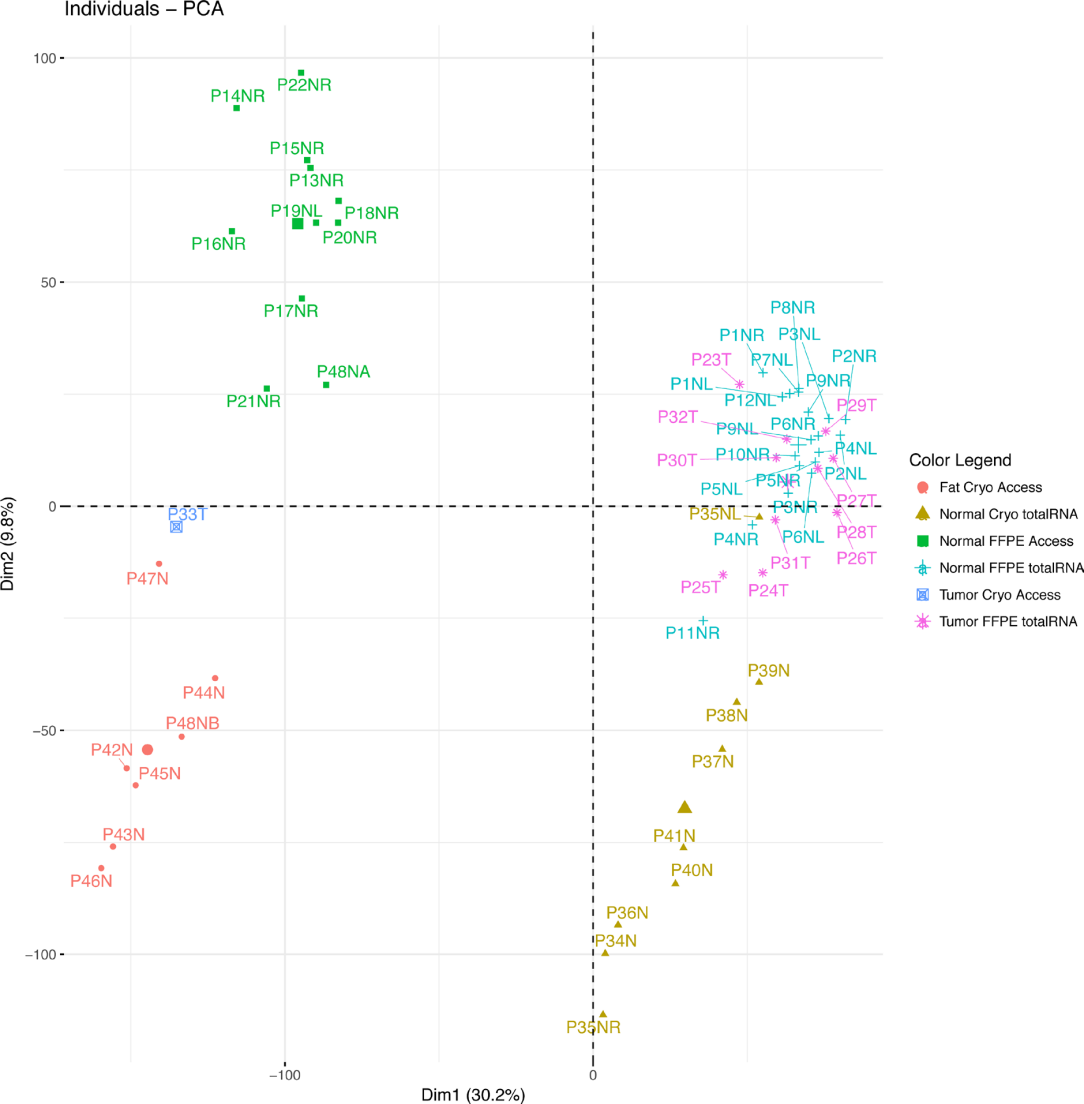
Principal component analysis based on gene expression The plot shows four distinct clusters of samples: The sequencing methods segregate on the first dimension. The adipocyte content or asservation methods segregate on the second dimension, where the pure adipocyte samples including the fat-rich sample half P48NB segregate in the lower left quadrant and the adipocyte-rich breast tissue samples in the lower right quadrant. The low-fat samples segregate on the upper half, including the fat-free sample half P48NA. Tumor segregation is not distinct.

Figure 2 shows a principal component analysis (PCA) plot of the samples based on read counts per million using all unfiltered 23686 genes. We included a previously published triple-negative breast cancer sample sequenced with TruSeq RNA Access [5]. The PCA plot shows very strong differences between total RNA and targeted RNA in the first principal component (Figure 2). Looking at the pairs of left and right breast tissue samples from the same patients, the PCA separated some pairs distinctly. In contrast, the PCA shows no separation between the tumor group and the normal group.

### Identification of genes with high expression variability in normal tissue

Next, we identified the genes with high expression variability in healthy normal tissue. Firstly, differential expression analysis was performed within each group by comparing each sample versus the remaining samples in the group. Using the statistical models in DESeq2 or Limma/voom, no significantly differentially expressed genes were detected. Secondly, using normalized gene expression (in read counts per million) to compare patient-matched left and right breast tissue samples, we found 2287 genes (FFPE) and 3745 genes (FF), that were differentially expressed with a log2 fold change of more than 2 in one or more patients (Supplementary Tables 12 and 13). Thirdly, expression variability within each group of normal data was computed on the basis of the normalized standard deviation or coefficient of variation (CV) of read counts per million. A CV of less than 0.30 was used as the threshold to separate low expression variability from high expression variability. This threshold corresponds to a maximal log2 fold expression change of 2 within a group of healthy normal samples, with a 95% confidence interval, for data that has a normal distribution. Table 1 summarizes the number of genes with low variability according to the CV threshold of 0.30. The PCA plot in Supplementary Figure 2 is based on gene expression where just these genes with low expression variability are considered.

### Identification of differentially expressed genes between FFPE and FF samples

Differential expression analysis was performed between the formalin fixed paraffin embedded samples and the fresh frozen samples that we sequenced using the total RNA method, in order to identify genes that were not highly differentially expressed between these two asservation methods. Pathway analyses based on the differentially expressed genes uncovered adipocyte signatures in the fresh frozen samples (Table 2). The bioinformatic finding of suspected fat cell content in the breast ductal tissue sample was confirmed to be true by the breast tissue biobank curator and surgeons.

**Table 2:** KEGG pathway analysis of differential expression between FFPE and FF (Kapa)

Name	pSize	NDE	pGFdr	pGFWER	Status
Neuroactive ligand-receptor interaction	154	50	3.2E-12	3.2E-12	Inhibited
PPAR signaling pathway	60	28	2.5E-10	5.1E-10	Inhibited
ECM-receptor interaction	77	23	9.6E-04	2.9E-03	Activated
Dilated cardiomyopathy	76	18	1.1E-02	4.7E-02	Inhibited
Cytokine-cytokine receptor interaction	186	31	1.1E-02	6.7E-02	Inhibited
Adipocytokine signaling pathway	64	13	1.1E-02	6.8E-02	Inhibited
Calcium signaling pathway	143	27	3.1E-02	2.2E-01	Inhibited
Basal cell carcinoma	47	12	4.5E-02	3.6E-01	Activated

pSize: Number of genes per pathway.

NDE: Number of differentially expressed genes per pathway.

pGFdr/pGFWER: False Discovery Rate and respectively Bonferroni adjusted global *p*-values.

Status: Direction in which the pathway is pertubated.

### Identification of differentially expressed genes between high-fat and fat-free samples

To identify gene expression that may be confounded by adipocyte content, two experiments were performed: (a) comparison of formalin fixed paraffin embedded normal healthy breast tissue samples with available fresh frozen normal healthy pure subcutaneous fat tissue samples, and (b) halving a fresh breast reduction tissue sample and comparing a macroscopically prepared fresh frozen half with the other half that was prepared by microdissection after formalin fixation and paraffin embedding. The sequencing experiment with these samples succeeded only with targeted RNA libraries, but not with total RNA libraries whose yield was too low for sequencing. The results are summarized in the Venn diagram in Supplementary Figure 3. Using a log2 fold change threshold of 2 and eliminating highly variable breast tissue genes with CV > 0.3, the comparison of breast tissue versus fat tissue resulted in 86 genes that were differentially expressed (Supplementary Table 14). 7 of these genes (8%) overlapped with the 3819 differentially expressed genes in the total RNA groups between formalin fixed paraffin embedded samples and fresh frozen samples (Supplementary Table 15). In the targeted RNA sequencing experiment with the halved samples, we filtered out the previously identified highly variable genes with CV > 0.3 in FFPE breast tissue TruSeq RNA Access libraries. After this filtering step, 116 genes were differentially expressed between the two halves (Supplementary Table 16). 55 of these genes (47%) overlapped with the differentially expressed genes between formalin fixed paraffin embedded breast tissue samples and the fresh frozen subcutaneous fat samples (Supplementary Table 15). In the principal component analysis plot based on unfiltered genes (Figure 2), the fresh frozen subcutaneous fat tissue samples are clearly separated from the formalin fixed breast tissue samples by the second principal component. In the same plot, the macroscopically prepared fresh-frozen ductal breast tissue sample “half” is clearly separated from its corresponding half that was microdissected, clustering with the subcutaneous fat tissue samples. The PCA plot in Supplementary Figure 4 is based on gene expression where only genes were considered that had low expression variability in healthy breast tissue and where the expression difference between high-fat and fat-free samples was no greater than the log2 fold change threshold of 2.

### Qualitative support of our RNA-Seq findings using GTEx data

Our own findings are supported by our analysis of GTEx expression data (Supplementary Figure 1) that were generated by the GTEx Project using Illumina TruSeq non-stranded mRNA (polyA) sequencing. Our PCA plot in Supplementary Figure 1 is based on read counts per million per gene. It shows that the GTEx mRNA data segregate from our whole transcriptome and targeted RNA-Seq clusters in a distinctly different location of the plot. Within the GTEx clusters, the fat samples cluster to the lower left of the healthy breast tissue samples. This supports our targeted RNA-Seq results where our pure fat samples cluster to the lower left of our healthy breast tissue cluster. The GTEx healthy breast sample cluster overlaps with the GTEx fat sample cluster, whereas our clusters are distinctly separated. Looking at the GTEx breast tissue gene expression data, there are only 2791 genes with a coefficient of variation less than 0.3 (Supplementary Table 17), and these do not overlap with our non-highly-variable genes. Looking at differentially expressed genes between GTEx breast and GTEx adipocyte data, 6366 are downregulated and 6624 upregulated (Supplementary Table 18), an overlap of 79% with our unfiltered differentially expressed genes.

### Breast cancer RNA-seq data

11 breast cancer tissue samples were included in the principal component plot (Figure 2). Figure 2 shows that the breast cancer samples are not clearly separated from the healthy normal samples, if all unfiltered genes are considered for the analysis. If at all, the tumor samples are separated from the healthy samples mainly by the second principal component.

To exemplify the use of the normal healthy breast tissue reference data for biomarker discovery studies, we performed differential expression analysis and matching of overexpressed genes to associated inhibitors between 10 tumor and 19 normal samples that came from formalin fixed paraffin embedded tissue and were all sequenced using the total RNA kit. Table 3 shows the overexpressed genes that were matched to inhibitors and annotated to indicate which gene may be an unreliable biomarker. To exemplify a personalized differential expression analysis, Table 4 shows the overexpressed genes of our previously published patient [5] that we associated with inhibitors or drugs and which we now annotated with gene expression variability in healthy normals.

**Table 3:** FDA-approved drugs associated to overexpressed genes in the group of 10 breast cancer samples (Roche Kapa total RNA-Seq)

Gene	log2FC	CV	ASG	TLV	Variability	Inhibitor
*TUBB3*	3.4	0.92	No	Yes	high	Ixabepilone *et al.*
*TOP2A*	3.1	1.84	No	Yes	high	Doxorubicin *et al.*
*RET*	2.7	1.72	No	No	high	Ponatinib *et al.*
*ROS1*	2.2	3.21	No	Yes	high	Crizotinib *et al.*

log2FC: log2-fold-change of expression between group of 10 cancer samples and group of 19 normal tissue samples. CV: coefficient of variation in FFPE healthy breast tissue samples (Roche Kapa). ASG: adipocyte signature gene. TLV: tissue location dependent variability of expression. Variability: variability of gene expression in healthy breast tissue (high: if CV > 0.3, or log2FC > 2 between tissue locations in same patient, or if adipocyte signature gene).

**Table 4:** Drugs associated to overexpressed genes in triple-negative breast cancer skin metastasis sample P33T (TruSeq RNA Access, targeted RNA-Seq)

Gene	log2FC	CV	ASG	TLV	Variability	Inhibitor
*RAD51*	5.0	0.56	Yes	No	high	Amuvatinib
*TOP2A*	4.9	0.50	No	Yes	high	Doxorubicin *et al.*
*AURKB*	4.5	1.32	No	Yes	high	Danusertib *et al.*
*AURKA*	4.3	0.59	No	No	high	Danusertib *et al.*
*CHEK1*	3.6	0.49	No	No	high	AZD7762
*CHEK2*	2.0	0.27	No	Yes	high	AZD7762
*NOTCH1*	2,0	0.27	Yes	No	high	RO4929097 *et al.*
*PARP1*	1.9	0.13	No	No	low	Olaparib *et al.*
*PIK3R2*	1.9	0.28	No	No	low	Apitolosib *et al.*
*RRM1*	1.8	0.14	Yes	No	high	Gemcitabine *et al.*
*SYK*	1.7	0.35	Yes	No	high	Fostamatinib
*CDK4*	1.6	0.17	No	No	low	Palbociclib *et al.*
*AKT1*	1.5	0.16	Yes	No	high	AZD5363 *et al.*
*MAP2K1*	1.4	0.23	Yes	No	high	Cobimetinib *et al.*
*ALK*	1.3	0.93	No	Yes	high	Crizotinib *et al.*
*ERBB3*	1.1	0.58	No	No	high	Osimertinib *et al.*

log2FC: log2-fold-change of expression between single triple-negative breast cancer metastasis and group of 10 normal tissue samples. CV: coefficient of variation in FFPE healthy breast tissue samples (TruSeq RNA Access)

Variability: variability of gene expression in healthy breast tissue (high: log2FC > 2). ASG: adipocyte signature gene. TLV: tissue location dependent variability of expression. Variability: variability of gene expression in healthy breast tissue (high: if CV > 0.3, or log2FC > 2 between tissue locations in same patient, or adipocyte signature gene). Inhibition of Poly [ADP-ribose] polymerase 1 (*PARP1* gene), Phosphatidylinositol 3-kinase regulatory subunit beta (*PIK3R2* gene) and Cyclin-dependent kinase 4 (*CDK4* gene) are therapeutic options suggested by tumor-vs-normal RNA overexpression versus the CV and low variability of RNA expression in the healthy tissue samples. The remaining overexpressed gene transcripts may be less confident RNA biomarkers due to their high variability in the healthy reference tissue between individuals, between tissue locations in the same individual, or due to potential confoundment by adipocyte content. Here, the skin metastasis was adipocyte-free.

### Immune repertoire reference data

Immune repertoire statistics are summarized in Supplementary Tables 6–9. In brief, the statistics depend most strongly on the sequencing method (total RNA vs targeted RNA sequencing). In particular, given the identical biological RNA samples, TCRα chain sequences are underrepresented in TruSeq RNA Access data by about an order of magnitude compared to Roche Kapa RiboErase. Additionally, B cell receptor sequences seem to be underrepresented in frozen samples compared to formalin samples. On average, the percentages of aligned immune repertoire reads were comparable in normal and cancer tissues, and one to two orders of magnitude lower in fat tissue. Individual cancer sample P27T shows a four-fold as high percentage of immune repertoire reads than other cancer samples and healthy tissue.

## DISCUSSION

The primary objective of our study was to generate RNA Seq expression reference data in healthy normal breast tissue for two RNA-Seq methods that are suitable for the small tissue sample amounts that are typically available from breast cancer patients. Next to the raw expression data, we provide Supplementary Tables 1–4 on those genes that appear to have a modest gene expression variability in healthy normal samples, i.e. with a coefficient of variation less than 0.3 which can approximately be interpreted as a log2 fold change lower than 2. We excluded genes with a coefficient of variation greater than 0.3 and we also identified and excluded over 3000 additional genes that varied more than 4-fold between the left and right healthy breast tissue sample pairs from the same patients. For the fresh frozen breast samples, we also excluded genes that may be subject to confounding by fat cell content.

We also generated breast cancer RNA-Seq data for demonstration purposes. The overall expression data in tumors were very similar to healthy breast tissue RNA-Seq data in the PCA plot (Figure 2). The biological difference between tumor and normal samples is clearly much smaller than the technological differences of sequencing or sampling methods. On an individual patient level, RNA-Seq may show more clearly than DNA-based panel sequencing which genes are overexpressed and which are not. Specifically for our patients, gene amplification data were available from a DNA-based test. However, the gene amplifications detected in patient tumors P24T and P26T (highlighted in light red in Supplementary Table 11) were not seen in our RNA-Seq expression data (Supplementary Table 10). This discrepancy is possibly explained by technological errors in the amplification detection method, or by biological deactivation (e.g. methylation or mutation of the promoter region) or biological downregulation. Of note, the DNA-based gene amplifications and the gene deletion detected in patient tumors P25T, P27T and P29T were clearly reflected in our RNA-Seq data. This suggests that amplifications detected by DNA-based methods alone may not be sufficiently actionable and that RNA-based follow-up should be considered to assess the expression change.

When comparing patient samples with our reference data, it is of highest importance to use the corresponding RNA sequencing method for the patient samples, i.e. Illumina TruSeq RNA Access or Roche Kapa RNA RiboErase. This is clearly seen in the PCA plots in Figure 2 and Supplementary Figure 1, and has also been reported by Li and colleagues [6]. It is also important to use the matching sample asservation method (formalin or freezing). Using formalin fixation and Roche Kapa RNA sequencing, 10259 genes are available for confident differential gene expression analysis (Table 1). With frozen samples and Roche Kapa RNA sequencing, 6719 genes are available. A fixation method mismatch does not appear to be a big problem when using our reference data: As can be seen in row 3 of Table 1, 6434 genes can be compared with our reference data if the method of fixation does not match, i.e. only 285 less than 6719. Of note, Table 1 shows that 36 breast cancer biomarkers may be unaffected by the fixation method. These are detailed in Supplementary Table 3, and include *CDK4* (associated with e.g. Palbociclib) and *MTOR* (associated with e.g. Everolimus).

We were alarmed by multiple highly significant adipocyte pathway signatures (Table 2) in the fresh frozen breast tissue samples and by the separate clusters of formalin and frozen samples in the PCA plot (Figure 2). Fat cell content was confirmed in the frozen samples, but formalin samples were not affected, as they were prepared from thin tissue sections after staining and microscope inspection. Therefore, we investigated the extent to which gene expression is confounded, using frozen pure fat tissue samples (Supplementary Table 14, Supplementary Figure 3). Eighty-six further genes that we had not already excluded showed significant differential expression and were excluded from the fresh frozen breast tissue reference data. Twenty-one of the well established breast cancer biomarkers, including *CDK4* and *MTOR* were unaffected by fat tissue in our sequencing data (Supplementary Table 5). In contrast to our unaffected *MTOR* RNA expression, mTORC1 and mTORC2 protein complexes are known to play an important role in adipocyte metabolism [7].

To follow up our observations of adipocyte signatures in breast samples, we analysed GTEx data from mRNA-sequencing of fat and breast tissue samples. Our principle component analysis (Supplementary Figure 1) confirmed the same adipocyte cluster location relative to the breast cluster location within the GTEx mRNA-Seq data as within our targeted RNA-Seq data. On the other hand, our breast samples cluster more distinctly than the GTEx samples and less genes are affected by high expression variability in our samples than in the GTEx data. Indeed, the GTEx breast cluster is so large that it entirely overlaps the GTEx adipocyte cluster, suggesting that the GTEx breast samples in the overlap region may consist mainly of adipocyte tissue. Accordingly, less than 3000 GTEx breast tissue genes show a coefficient of variability less than 0.3, compared to between about 7000 and 10,000 genes in our here presented breast tissue reference data for targeted RNA-Seq and total RNA-Seq, respectively.

Of note, the GTEx standard operating procedure for biospecimen collection (https://biospecimens.cancer.gov/resources/sops/docs/GTEx_SOPs/BBRB-PR-0004%20GTEx%20Tissue%20Processing%20Procedure.pdf) provides guidance on adipocyte removal: “For specimens embedded in adipose tissue (e.g., arteries, nerve, adrenal, pancreas, and skeletal muscle) dissect/tease off peripheral fat as thoroughly as feasible without damaging the target tissue using ‘blunt’ technique and following tissue planes”. This procedure was used when we collected our fresh frozen breast tissue samples. From our own sampling and RNA-Seq results we conclude that FFPE sampling enables the potentially confounding adipocytes to be removed more effectively than fresh breast tissue sampling on the surgeon's table.

We also provide reference data on B- and T-cell receptor repertoires in our healthy breast tissue samples and our tumor samples in Supplementary Tables 6–9. These immune repertoire data should be used with samples that are processed with matching fixation and sequencing methods. The immune repertoire statistics are no validated biomarkers. They are currently subject to research. Possibly they may become validated biomarkers, helping the clinician to decide whether sufficient T-cells have infiltrated the tumor tissue for a PD1-/PD-L1 checkpoint inhibitor, or whether additional immunological strategies are indicated, such as those that increase T-cell priming, proliferation and penetration into the tumor tissue.

In conclusion, our gene expression and immune repertoire reference data aim to help identify true aberrations and ultimately guide experimental breast cancer drug selection in last-line patients with or without patient-matched normal tissue.

## MATERIALS AND METHODS

### Patients and tissue samples

Tissue samples were obtained from the biobanks at the Avera Cancer Institute and at the University Hospitals Schleswig-Holstein, Kiel, Department of Gynaecology and Obstetrics and Department of Dermatology. All patients provided informed consent and the study was performed according to internal ethics review board approvals at the Avera Cancer Institute and at University Hospitals Schleswig-Holstein, respectively.

19 normal FFPE breast ductal tissue samples were obtained at Avera, with seven pairs of left and right breast and five from either left or right breast from a total of 12 individuals. 10 breast cancer FFPE tissue samples were collected at Avera. Nine breast ductal normal FF (liquid nitrogen) tissue samples were collected at the Department of Gynaecology and Obstetrics, Schleswig-Holstein from breast reduction operations. One pair of FF left and right healthy normal breast ductal tissue samples were from the same breast reduction patient. 1 healthy breast ductal tissue sample was collected at the Department of Gynaecology and Obstetrics, Schleswig-Holstein from a breast reduction patient and rapidly halved. From the first half, fat tissue was macroscopically removed by the surgeon and the sample was snap frozen in liquid nitrogen. The second half was quickly transported to the pathologist at 4°C, fixed in formalin, embedded in paraffin, and stained with hematoxylin and eosin. Ductal epithelial cell regions in this FFPE sample were marked under a microscope for laser microdissection. Six subcutaneous fat tissue healthy samples were collected in RNA*later* RNA Stabilization Reagent (Cat # 76104, Qiagen, Hilden, Germany) at the Department of Dermatology, Schleswig-Holstein, kept over night without freezing, and then frozen at −80°C.

### RNA isolation

Deparaffinization of FFPE samples was performed using 1ml of xylene at 50°C for 3 min followed by two washes with 100% ethanol. Ethanol was removed by pipet and samples were then dried at room temperature to completely remove residual ethanol. The remaining pellet was then extracted. RNA from the Avera Cancer Institute's FFPE samples was isolated using the Maxwell RSC RNA FFPE Kit (Cat # AS1440, Promega, Madison, WI, USA) on the Maxwell automated system (Cat # AS4500, Promega, Madison, WI, USA) according to the manufacturers' protocol. This RNA was quantitated using the Qubit instrument and RNA HS kit (Invitrogen). All samples were treated with DNAse according to protocol and subsequently purified using AMPure RNA XP Clean beads (Agencourt Bioscience Corp., Austin, TX, USA). RNA from FF breast tissue was isolated at the Institute of Clinical Molecular Biology with the mirVana™ miRNA Isolation Kit with phenol (Cat # AM1560, ThermoFisher, Waltham, MA, USA) according to the manufacturer's protocol. RNA from fat tissue samples was isolated at the Department of Dermatology using the Qiagen AllPrep DNA/RNA Mini Kit (Cat # 80204, Qiagen, Hilden, Germany), according to the manufacturer's protocol. RNA from the single German FFPE breast tissue sample was isolated at the Institute of Clinical Molecular Biology using the RecoverAll FFPE kit (Cat # AM1975, Ambion/Invitrogen, Carlsbad CA, USA) according to manufacturer's protocol. The RNA from the German samples was quantitated with Qubit (ThermoFisher, Waltham, MA, USA) and TapeStation 2200 (Agilent Technologies, Waldbronn, Germany).

### Next-generation sequencing

For library preparation, the Illumina RNA Access Library Prep Kit (Cat. No. RS-301-2001, Illumina, San Diego, CA, USA) was used with 40 ng of FFPE RNA or 50 ng of FF RNA, respectively. At time of writing the kit has been split into three kits and renamed to TruSeq RNA Exome, consisting of TruSeq RNA Library Prep for Enrichment (Cat. No. 20020189), TruSeq RNA Enrichment (Cat. No. 20020490), Exome Panel (Cat. No. 20020183). The libraries were prepared according to the manufacturer's protocol with the following change for the FFPE samples: the hybridization/capture was performed individually instead of as pooled samples after the first PCR step.

For library preparation, the KAPA Stranded RNA-Seq Library Prep Kit (Roche) was used with 100 ng of FFPE RNA according to the manufacturer's protocol.

Libraries were quantified using the Qubit instrument and DNA HS kit (Invitrogen) for the FFPE samples, and Qubit and TapeStation for the FF samples. Library concentrations were adjusted to 1nM and pooled for multiplex sequencing. Pooled libraries were denatured and diluted to 7.5 pM and clonally clustered onto a NextSeq 500 High Output sequencing flow cell. The clustered flow cells were sequenced on the Illumina NextSeq 500 Platform to 75bp paired end reads.

### Primary data analysis

Processing was performed using a custom workflow implemented in the pipelining framework OmicsPipe [8]. Adapter sequences were trimmed and low quality reads removed using BBDuk version 34.46 from the BBMap suite (https://sourceforge.net/projects/bbmap/) with parameters: minlen = 25 qtrim = rl trimq = 10 ktrim = r k = 25 mink = 11 hdist = 1 overwrite = true tbo = t tpe = t. Quality of raw reads was assessed using FastQC (https://www.bioinformatics.babraham.ac.uk/projects/fastqc/) version 0.11.2. Mapping of RNA and generation of gene counts was done using STAR [9] aligner version 2.4.2a against human reference hg19 and using Refseq hg19 gene annotation. RNA-Seq based count data is available in Supplementary Table 10.

### Differential expression and pathway analysis

Main analysis were undertaken using R version 3.4. RNA-Seq count data were processed and differential expression analysis performed using limma/voom methodology [10] and DESeq2 [11]. Pathway analysis was performed using signaling pathway impact analysis (SPIA) [12]. Enrichment analysis was performed using g:Profiler [13]. In all statistical tests, an effect was considered as statistically significant if the *P*-value of its corresponding statistical test was ≤ 5%. Differential expression between patient-matched pairs of single samples (left vs right breast, FFPE half vs FF half) was additionally computed using STAR counts per million instead of the statistical tests in limma or DESeq2. Cancer biomarkers (Supplementary Table 19) were obtained from the Cancer Genome Interpreter (https://www.cancergenomeinterpreter.org/biomarkers). Breast cancer associated genes (Supplementary Table 20) were obtained using GLAD4U.

### Drug selection based on overexpressed genes

Drug selection was performed on differentially overexpressed genes with log2 fold change greater than 1.5. We queried DGIdb API V2 [14] with the following parameters: interaction type ‘inhibitor’ (antagonist, antibody, blocker, inhibitor, suppressor, allosteric modulator, adduct, binder, immunotherapy, inhibitory allosteric modulator, inverse agonist, vaccine), anti neoplastic: true, clinically actionable: true and source trust level: expert curated.

### Immune repertoire fraction of reads and clonotype count estimate

The number of sequences per sample was harmonized by downsampling the FASTQ files to the number of reads in the smallest file. Sequences were processed using the immune repertoire detection software MiXCR [15], version 2.1.1. Briefly, sequences were aligned against human immune repertoire reference including V, D, J and C genes of TCR αβ and γδ chains and BCR/immunoglobulin (IG) heavy (IGH) and light (IGK, IGL) chains. Where present, complementary determining region 3 (CDR3) sequences were extrapolated and worked as identifier sequences for clonotype clustering.

Absolute number and percentages of aligned reads were calculated for each immune repertoire chain (both TCRs and BCRs) as well as the number of final clonotypes identified.

## SUPPLEMENTARY MATERIALS FIGURES AND TABLES







## Abbreviations

Access: Illumina TruSeq RNA Access
BCR: B-cell receptor
BrCa: breast cancer
CV: coefficient of variation, normalized standard deviation
DE: differentially expressed/differential expression
DGIdb API: Drug-Gene Interaction database application programming interface
FF: fresh frozen
FFPE: formalin fixed paraffin embedded
IGH: BCR/immunoglobulin heavy chain
IGK and IGL: BCR/immunoglobulin light chains (κ and λ)
Kapa: Roche Kapa RNA RiboErase
PD1: Programmed cell death protein 1
PD-L1: Programmed cell death ligand 1
TCR: T-cell receptor
TRA and TRB: T-cell receptor α and β loci
