# Supplementary material for: RNA based individualized drug selection in breast cancer patients without patient-matched normal tissue
Source: Oncotarget. 2018 Aug 17;9(64):32362–72. doi: 10.18632/oncotarget.25981 (PMC6122351; doi:10.18632/oncotarget.25981)
Supplement: Supplementary file 1 [file oncotarget-09-32362-s001.pdf]

RNA based individualized drug selection in breast cancer patients without patient-matched normal tissue

SUPPLEMENTARY MATERIALS

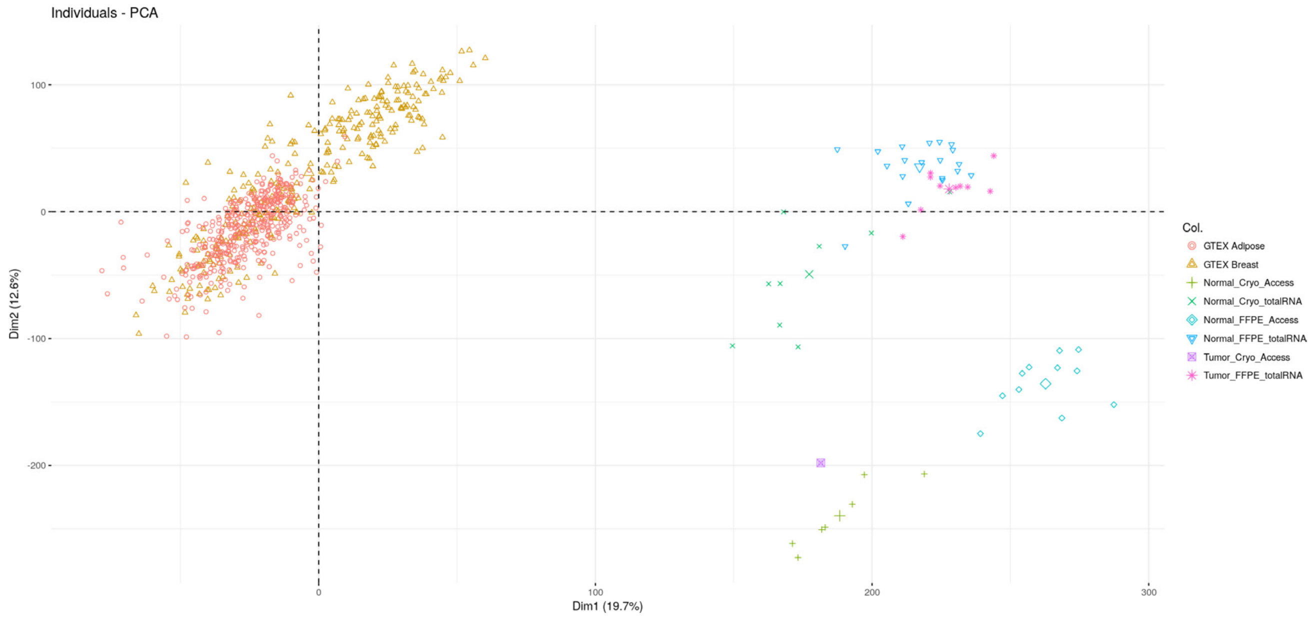

Supplementary Figure 1: Principal component analysis of our data compared to GTEx data. Based on genes that are shared in the GTEx gene expression data annotations and our annotations.

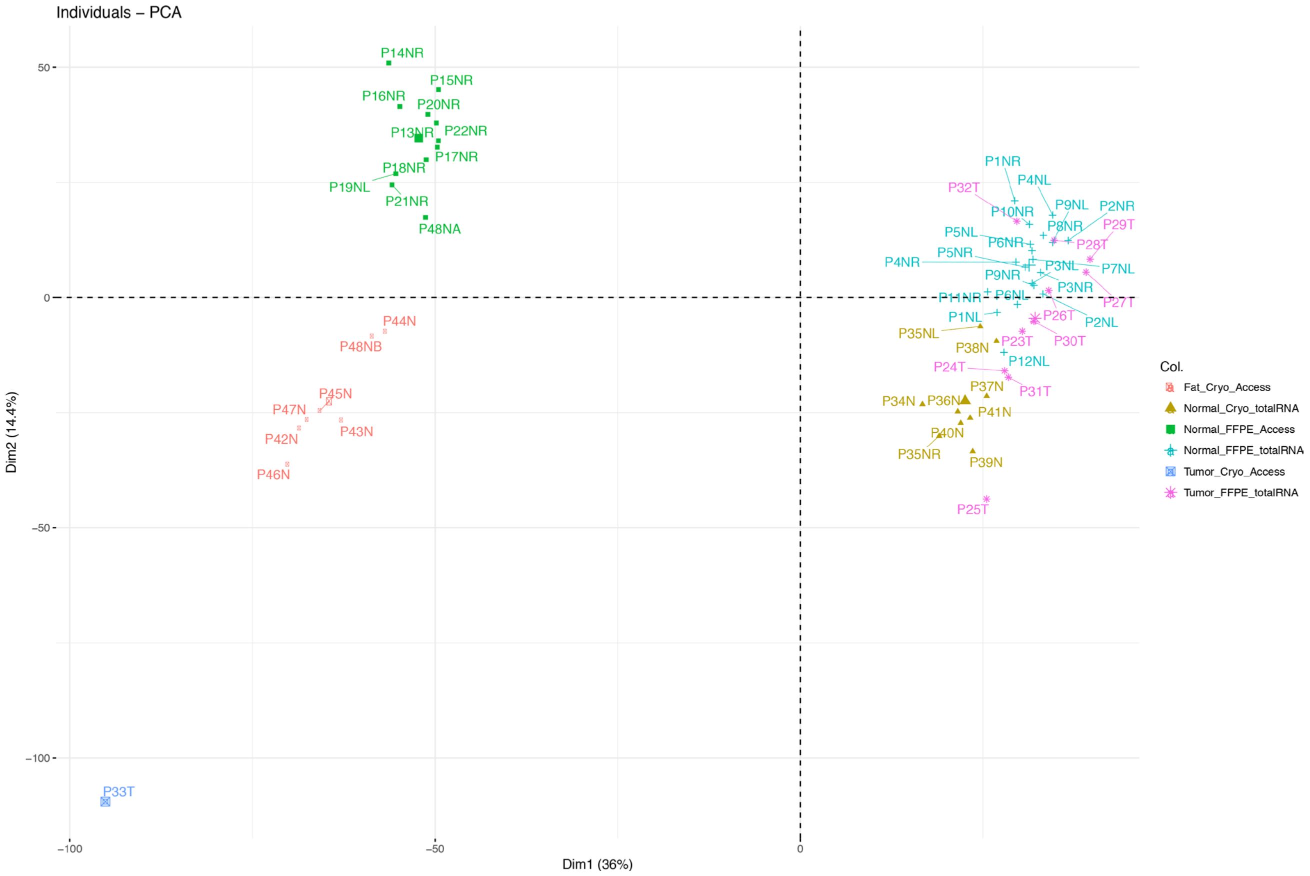

Supplementary Figure 2: Principal component analysis of our data based on expression of genes that are not highly-variable genes.

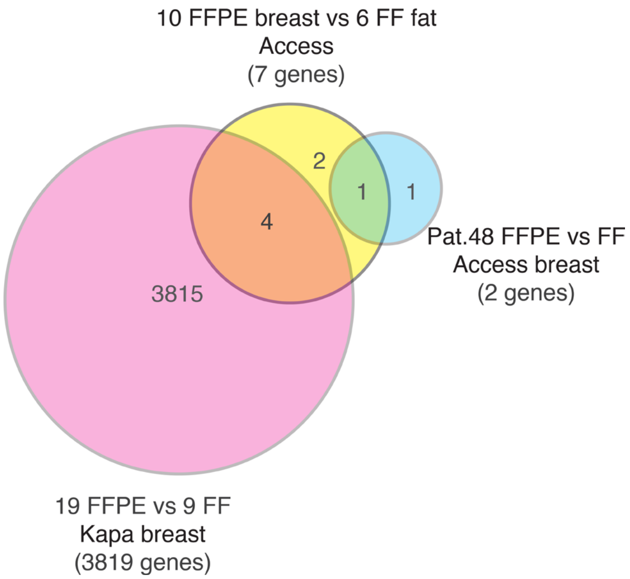

Supplementary Figure 3: Venn diagram showing number of fat associated genes in our data.

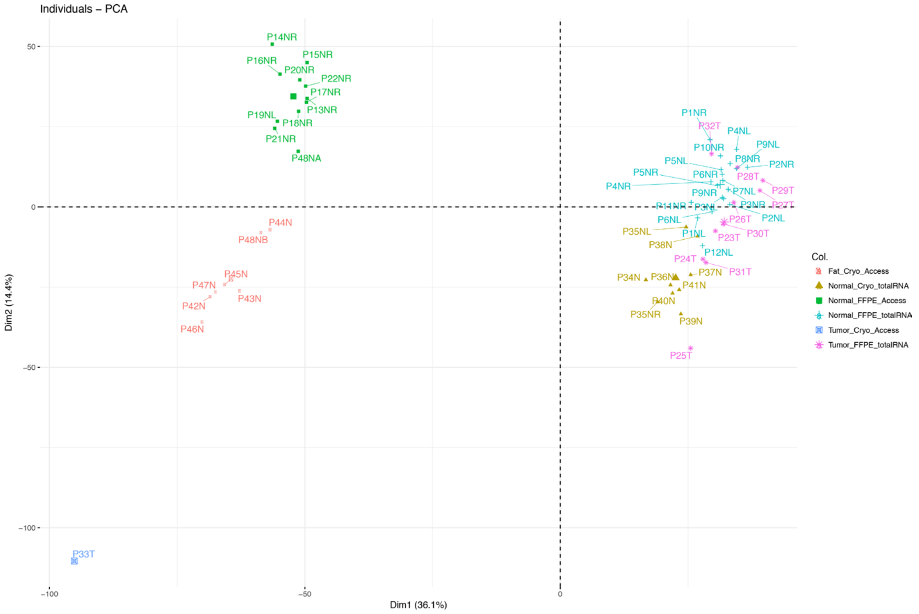

Supplementary Figure 4: Principal component analysis of our data based on expression of genes that are not highly-variable genes and that are not adipocyte signature genes.

Supplementary Table 1: Genes with low expression variability in healthy breast ductal tissue (Roche Kapa, Formalin Fixed Paraffin Embedded tissue). See Supplementary_Table_1

Supplementary Table 2: Genes with low expression variability in healthy breast ductal tissue (Roche Kapa, Fresh Frozen tissue, CVFF <= 0.3). See Supplementary_Table_2

Supplementary Table 3: Genes with low expression variability in healthy breast ductal tissue and low differential expression between FFPE and FF tissue (Roche Kapa). See Supplementary_Table_3

Supplementary Table 4: Genes with low expression variability in healthy breast ductal tissue (Illumina TruSeq RNA Access, Formalin Fixed Paraffin Embedded tissue). See Supplementary_Table_4

Supplementary Table 5: Genes with low differential expression between epithelial breast duct tissue (FFPE) and pure fat tissue (FF), and with low expression variability in healthy breast ductal tissue (Illumina TruSeq RNA Access). See Supplementary_Table_5

Supplementary Table 6: Immune repertoire statistics for healthy epithelial breast tissue and for breast cancer, using FFPE samples and Roche Kapa RiboErase total RNA-Seq. See Supplementary_Table_6

Supplementary Table 7: Immune repertoire statistics for healthy epithelial breast tissue, using fresh frozen samples and Roche Kapa RiboErase total RNA-Seq. See Supplementary_Table_7

Supplementary Table 8: Immune repertoire statistics for healthy epithelial breast tissue and for breast cancer, using FFPE samples and Illumina TruSeq RNA Access targeted RNA-Seq. See Supplementary_Table_8

Supplementary Table 9: Immune repertoire statistics in a breast cancer metastasis (FF), healthy fat tissue (FF), healthy epithelial breast tissue (FFPE vs FF), sequenced with Illumina TruSeq RNA Access targeted RNA-Seq. See Supplementary_Table_9

Supplementary Table 10: Counts per Million (CPM) for all breast, fat and breast cancer samples sequenced at Avera Cancer Institute and Institute of Clinical Molecular Biology (Forster *et al.* 2018). See Supplementary_Table_10

Supplementary Table 11: Sequencing reads per sample. See Supplementary_Table_11

Supplementary Table 12: Genes in patient-matched left and right FFPE breast tissue samples that were differentially expressed with a log2 fold change of more than 2 in one or more patients (P1, P2, P3, P4, P5, P6, P9). See Supplementary_Table_12

Supplementary Table 13: Genes in patient-matched left and right FF breast tissue samples that were differentially expressed with a log2 fold change of more than 2 in patient P35. See Supplementary_Table_13

Supplementary Table 14: Gene expression confounded by adipocytes: Significantly differentially expressed genes in FF pure fat tissue vs FFPE normal breast tissue (fat-free), TruSeq RNA Access sequencing. See Supplementary_Table_14

Supplementary Table 15: For Venn Diagram: Significantly differentially expressed genes in three comparisons, and their overlaps. See Supplementary_Table_15

Supplementary Table 16: Significantly differentially expressed genes in halved normal breast sample P48 FFPE (fat free) vs FF (some fat content). See Supplementary_Table_16

Supplementary Table 17: GTEx breast tissue - genes with low expression variability in healthy breast tissue (mRNA sequencing, fresh frozen samples). See Supplementary_Table_17

Supplementary Table 18: Differential gene expression between GTEx breast tissue and GTEx subcutaneous fat tissue. See Supplementary_Table_18

Supplementary Table 19: Cancer Biomarker database from Cancer Genome Interpreter (December 5 2017). See Supplementary_Table_19

Supplementary Table 20: BrCa associated genes from GLAD4U (December 4 2017). See Supplementary_Table_20
